# Clinical experiences of 109 children with foreign body ingestion: a retrospective study from Kunming, China

**DOI:** 10.3389/fped.2026.1771328

**Published:** 2026-03-10

**Authors:** Zhuoheng Li, Cuicui Yang, Jintao Duan, Jiahui Fang, Jun Chen, Shuli He, Juan Li

**Affiliations:** 1 Department of Gastroenterology, Kunming Children’s Hospital, Kunming, China; 2 Department of Pediatrics, Baoshan People’s Hospital, Baoshan, China

**Keywords:** batteries, coins, foreign body ingestion, pediatric, retrospective analysis

## Abstract

**Aim:**

This study was designed to assess the types and locations of foreign bodies ingested by pediatric patients, investigate the complications associated with these ingestions, and explore the demographic factors influencing the incidence of specific foreign body types and outcomes.

**Methods:**

A retrospective analysis was conducted on 109 pediatric patients who presented with foreign body ingestion due to foreign body ingestion (FBI). Data were collected on patient demographics (age, sex, body weight, and living environment), types of ingested materials (metal, plastic, magnetic), and anatomical locations of foreign bodies (esophagus, stomach, duodenum). The occurrence of complications, such as mucosal erosion, congestion, and edema, was also recorded. Descriptive statistics was used to analyze relationships between variables.

**Results:**

The majority of ingested foreign bodies were metallic, with coins being the most common object, followed by batteries and other metals. Plastic materials and magnetic beads were also noted. The most frequent locations for foreign body impaction were upper esophagus and stomach. Complications such as mucosal erosion and congestion were observed.

**Conclusion:**

This study highlights the prevalence of metallic foreign bodies, particularly coins, in pediatric FBI cases. The upper esophagus and stomach were the most common locations for impaction.

## 1 Introduction

Foreign body ingestion (FBI) in children is a common pediatric emergency, with the majority of cases involving children aged less than 5 years (1). The materials ingested by children are often household items or toys, which can cause various degrees of harm to the digestive system (2). Complications, including esophageal erosions, mucosal congestion, and edema, may occur, leading to symptoms such as pain, difficulty swallowing, and vomiting (3). These complications not only result in significant morbidity but also make FBI in children a growing public health concern.

Despite the widespread occurrence of pediatric FBI, existing studies show considerable heterogeneity in the types of ingested materials, anatomical locations, and related complications (4, 5), highlighting the need for additional data from diverse populations and regions from China. Although there are some studies available regarding the clinical management of FBI, it often focuses on isolated aspects, such as endoscopic retrieval or complications resulting from certain materials (6, 7). Most studies have either emphasized the treatment of specific foreign bodies or the outcomes of particular types of complications (8, 9), leaving a gap in the understanding of the broader spectrum of FBI-related injuries in the pediatric population.

The purpose of this study is to examine the types of foreign bodies ingested by pediatric patients, their anatomical locations within the digestive system, and the complications arising from their ingestion. The findings of this study will contribute to the growing body of literature on FBI in children and guide clinicians in the management of these cases.

## 2 Materials and methods

### 2.1 Study design

This was a retrospective observational study conducted at Kunming Children's Hospital, with an aim to investigate the types of foreign bodies ingested by children, the anatomical locations of these foreign bodies, and the associated complications. A total of 109 pediatric patients (66 males, 43 females) were included, who presented with FBI at the hospital over a period from Jan. 2021 to Oct. 2024. All included patients were younger than 12 years of age, and those with incomplete medical records were excluded from the study. The study was conducted in accordance with both the Declarations of Helsinki and approved by the hospital's institutional review board (IRB) [approval No.: IEC-C-008-A07]. The individual consent for this retrospective analysis was waived.

### 2.2 Demographic data

Demographic information, including patient age, sex, body weight, and living environment (urban or rural), was collected from the hospital's electronic medical records. Age was categorized into five groups: 0–3 years, 3–6 years, 6–9 years, 9–12 years, and ≥12 years.

### 2.3 Types of foreign bodies

Foreign bodies ingested by the patients were categorized into six types: metal (coins, batteries, and other metals), plastic (in-game currency, lollipop sticks), magnetic beads, and other miscellaneous materials (e.g., food mass, bone, and fish bones). The types of materials ingested were determined based on clinical presentation and radiographic findings.

### 2.4 Foreign body locations

The anatomical location of the ingested foreign bodies was documented based on radiographic imaging and endoscopic findings. Foreign bodies were classified into five anatomical regions: upper esophagus, middle esophagus, lower esophagus, stomach, and duodenum. The locations were determined by x-ray or endoscopic retrieval procedures, with the upper esophagus defined as the area from the pharynx to the cricopharyngeus muscle, the middle esophagus from the cricopharyngeus to the level of the tracheal bifurcation, and the lower esophagus as the segment from the bifurcation to the gastroesophageal junction.

### 2.5 Complications

Post-ingestion complications were classified into four groups based on the severity of symptoms: congestion, edema, erosion, and congestion combined with edema and erosion. Congestion was defined as the presence of visible mucosal swelling and erythema, while edema was diagnosed based on radiographic or endoscopic evidence of tissue fluid accumulation. Erosion was defined as superficial tissue damage caused by the foreign body. Clinical management and outcomes of each complication were recorded, and any surgical intervention required was also documented.

### 2.6 Removal techniques

Foreign bodies were removed using standard pediatric foreign body forceps, which were the primary tool for retrieval in the majority of cases. For foreign bodies that could not be retrieved with forceps, alternative tools such as snares, baskets, loop extractors, or magnets were employed. The method of retrieval was determined by the size, shape, and location of the foreign body. All patients underwent removal procedures under general anesthesia, depending on the size and location of the ingested foreign body.

### 2.7 Data collection

Demographic data, foreign body characteristics, locations, complications, and removal techniques were all collected and analyzed. Descriptive statistics, including means, standard deviations, and percentages, were used to summarize the data.

## 3 Results

### 3.1 Demographic data

Totally, 109 pediatric patients (male: 66; female: 43) were included in this retrospective analysis. All the patients presented to the Emergency Department and Outpatient Department, and underwent FBI removal immediately. The mean age was 44.36 ± 29.23 months. The body weight was 16.34 ± 8.11. Among 109 children, 64 live in urban areas and 45 live in rural areas. FBIs most frequently involved children aged less than 6 years of age, in which those aged less than 3 years accounting for 44.95% and those aged 3–6 years accounted for 40.37%. Compared with the girls, boys showed a higher incidence of FBI with an incidence of 60.55% (Table 1).

**Table 1:** Patients characteristics.

Variables	Patient number	Percentage (%)
Sex		
Male	66	60.55%
Female	43	39.45%
Age		
0–3 years	49	44.95%
≥3–6 years	44	40.37%
≥6–9 years	10	9.17%
≥9–12 years	5	4.59%
≥12 years	1	0.92%
Body weight		
<10 kg	6	5.50%
≥10–20 kg	81	74.31%
≥20–30 kg	15	13.76%
≥30–40 kg	1	0.92%
≥40 kg	6	5.50%
Living environment		
Urban	64	58.72%
Rural	45	41.28%

### 3.2 Types of ingested objects

**Figure 1:**
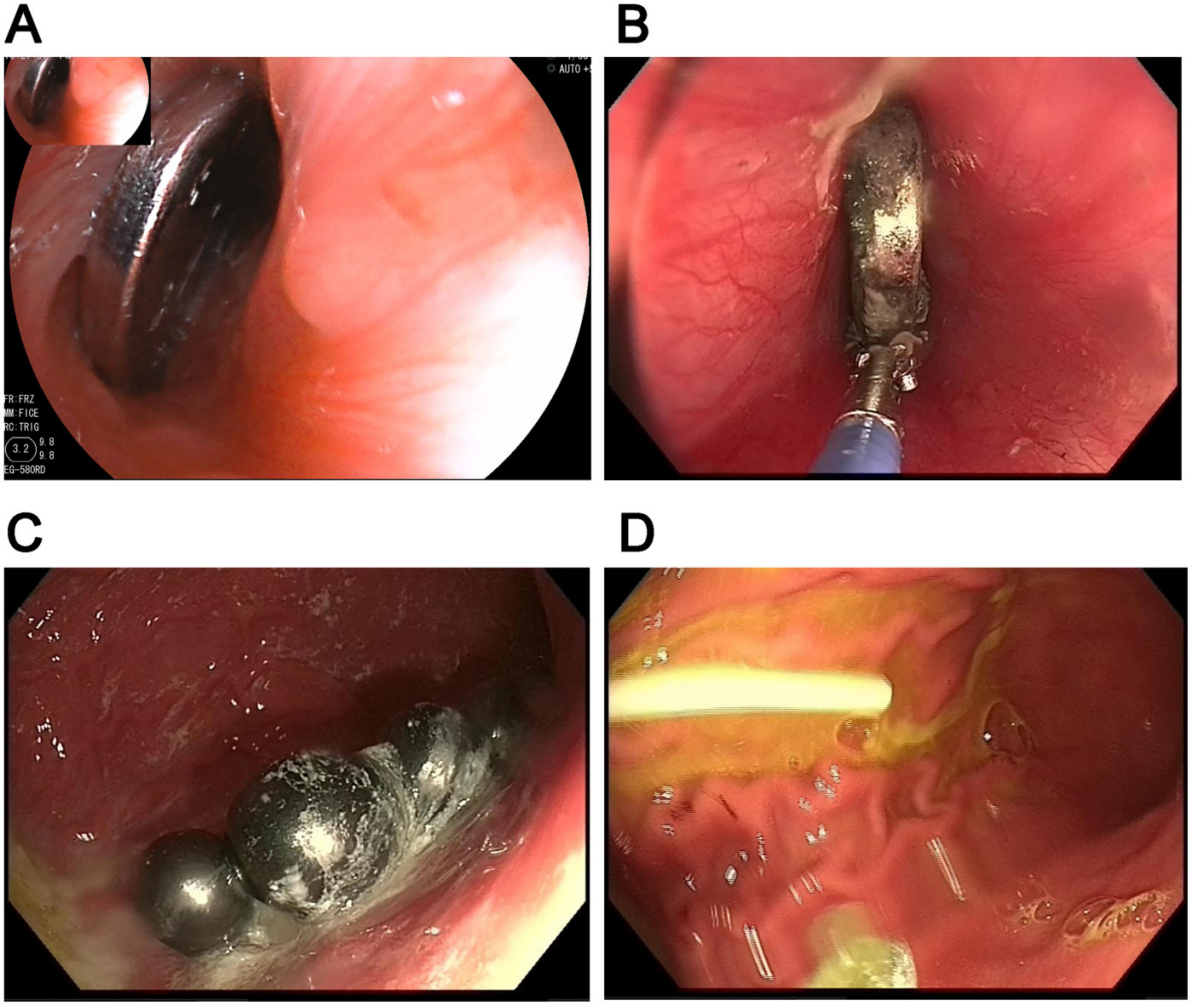
Types of FBI. **(A)** coin; **(B)** battery; **(C)** magnetic bead; **(D)** lollipop stick.

Most patients (81) showed ingestion of metal objects, including coins (*n* = 67), and batteries (*n* = 8), and others such as strew (*n* = 6). Fourteen showed ingestion with plastic materials, including 4 with ingestion of in-game currency, and 10 ingested sharp FBI, such as fish bone, hairpin and straw, and lollipop stick. Four patients showed ingestion of magnetic beads. The other 10 patients ingested objects were tablet, food mass, bone and fish bone (Table 2, Figure 1).

**Table 2:** Summary of materials in the patients with ingestion of foreign bodies.

Materials	Patient number	Percentage
Metal		
Coins	67	61.47%
Batteries	8	7.33%
Other metals	6	5.50%
Plastic		
In-game currency	4	3.67%
Lollipop stick, straw	10	9.17%
Magnetic beads	4	3.67%
Others	10	9.17%

### 3.3 FBIs locations

**Figure 2:**
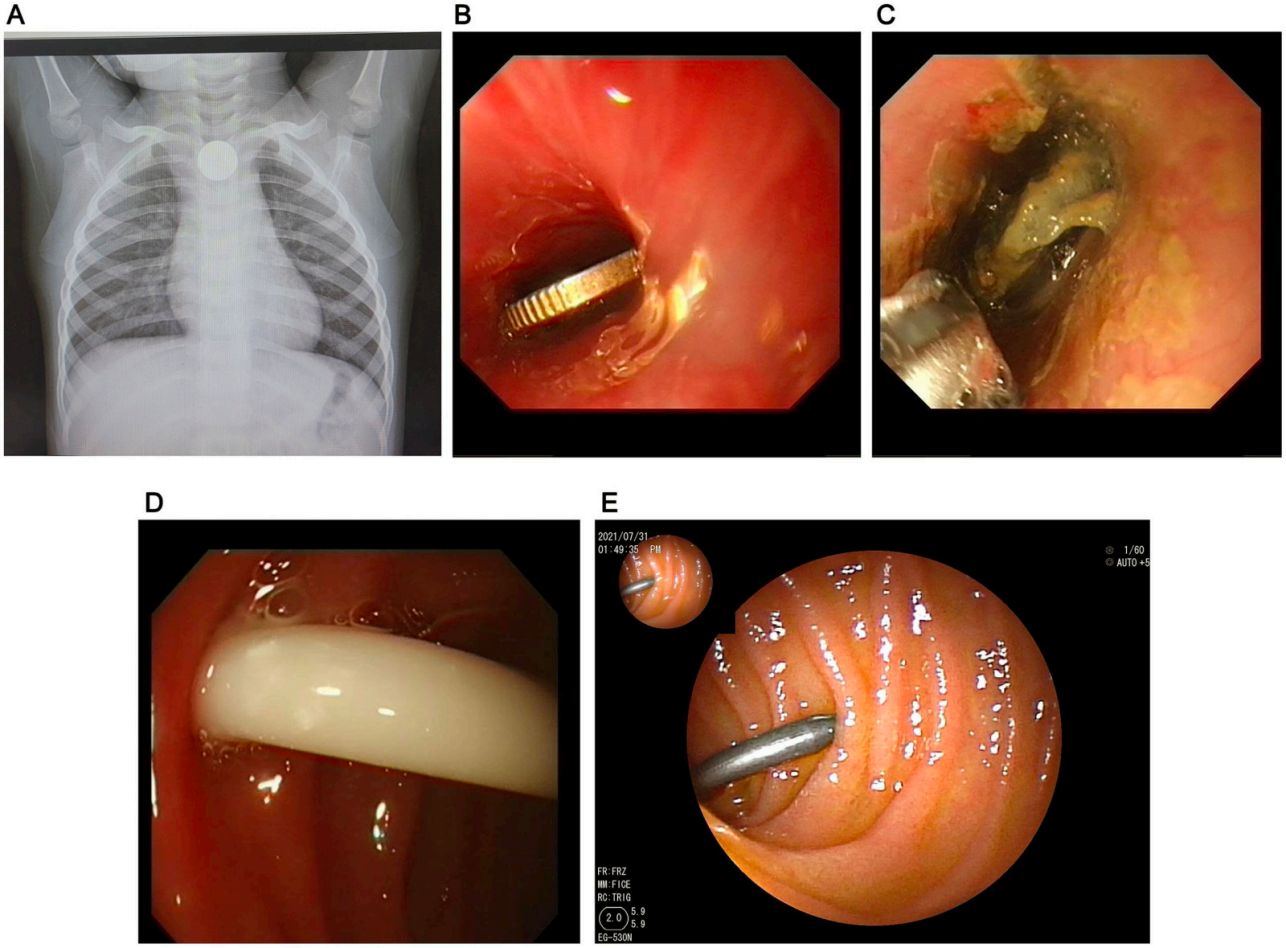
Locations of the FBI. **(A)** Location of a battery in the upper esophagus; **(B)** A coin in the middle esophagus; **(C)** A battery in the lower esophagus; **(D)** A lollipop stick in the stomach; **(E)** A screw in the duodenum.

The FBIs locations included upper (*n* = 38), middle (*n* = 11) and lower esophagus (*n* = 11), stomach (*n* = 46), and duodenum (*n* = 3). The majority of foreign bodies were located in the stomach (42.2%), followed by the upper esophagus (34.86%). The types of materials ingested varied across regions, with metal objects (e.g., coins and batteries) being the most common in the upper esophagus, while a greater variety of materials, including coins, batteries and plastics, were found in the stomach. The lower esophagus also contained a significant number of metallic objects. Foreign bodies in the duodenum were rare (2.75%), with the majority consisting of small metal or plastic items (Table 3; Figure 2).

**Table 3:** Summary of FBI locations.

FBI location	Patients number	FBI type					
		Coin	Battery	Other metals	Plastic	Magnetic	Others
Upper	38 (34.86%)	29	2	2	2	/	3
Middle	11 (10.09%)	7	/	/	2	/	2
Lower	11 (10.09%)	7	/	2	/	/	2
Stomach	46 (42.20%)	20	6	4	9	4	3
Duodenum	3 (2.75%)	2	/	/	1	/	/

### 3.4 Complications

Among the 109 cases, 43 patients showed complications after ingestion of FBI (Table 4). The most frequent complications included congestion, edema, and erosion. 7 showed congestion, 19 showed erosion. In addition, 5 showed congestion combined with erosion, and 12 showed congestion, combined with edema and erosion. Metal is the most common material ingested across all groups, followed by plastic. Magnetic and other materials are less common. The upper esophagus is the most frequently involved region, followed by the stomach. Some cases were found in the middle and lower esophagus, with no cases in the duodenum.

**Table 4:** Summary of complications after FBI.

Variables	Age, year	Male	FBI type				FBI location				
			Metal	Plastic	Magnetic	Others	Upper	Middle	Lower	Stomach	Duodenum
Erosion (*n* = 19)	3.53 ± 2.37	12 (63.16%)	14 (73.68%)	4 (21.05%)	/	1 (5.26%)	13 (68.42%)	2 (10.53%)	3 (15.79%)	1 (5.26%)	/
Congestion (*n* = 7)	4.26 ± 3.06	5 (71.42%)	5 (71.42%)	2 (28.57%)	/	/	1 (14.29%)	/	2 (28.57%)	4 (57.14%)	/
Congestion and erosion (*n* = 5)	4.35 ± 2.78	3 (60%)	4 (80%)	/	/	1 (20%)	1 (20%)	1 (20%)	2 (40%)	1 (20%)	/
Congestion, combined with edema and erosion (*n* = 12)	3.06 ± 1.63	7 (58.33%)	7 (58.33%)	2 (%)	1 (8.33%)	2 (16.67%)	3 (25%)	5 (41.67%)	1 (8.33%)	3 (24%)	/

### 3.5 Methods for the FBIs removal

The majority of patients (*n* = 100) underwent foreign body removal using foreign body forceps. The remaining cases had the foreign body removed using a snares (3 cases), a basket (2 cases), a loop extractor (2 cases), and magnets (2 cases).

## 4 Discussion

FBI is a frequent pediatric emergency, and while the clinical outcomes of these incidents have been well-documented, detailed comparisons across different studies remain limited (10, 11). The findings in our cohort align with and expand upon existing literature by shedding light on age-related trends, material types, and anatomical regions affected by foreign bodies in children.

Our study revealed that children under six years of age were most susceptible to foreign body ingestion, with a significant proportion (44.95%) aged 0–3 years, mirroring trends seen in previous studies (12, 13). Younger children, particularly toddlers, are in the critical age range where mouthing objects is a primary means of exploring the environment. This behavior, combined with underdeveloped swallowing reflexes and motor coordination, makes them especially vulnerable. Similarly, a study by Besharah et al. (14) found that children under the age of four accounted for over 50% of FBI cases, supporting the view that young age is a significant risk factor (14). The male predominance (60.55%) in our cohort is consistent with multiple studies, including a study by Mantegazza et al. (9), which reported that boys were more likely to ingest foreign bodies due to their higher physical activity levels and risk-taking behaviors.

A striking finding from our study is the predominance of metal objects, particularly coins, in the cases of FBI. Coins accounted for 61.47% of ingestions, followed by batteries (7.33%) and sharp objects (such as fish bones and hairpins). This distribution is consistent with findings from Lee (15) (15), who reported that coins were the most commonly ingested objects in children, often due to their widespread availability and appeal. Coins tend to lodge in the upper esophagus, where they can cause obstruction and mucosal injury. Button batteries, while less frequent (7.33% in our study), present a unique and often more dangerous risk, leading to severe complications like mucosal burns, perforation, or even death if not promptly removed (16). In a previous study, the Paediatric Surgery TraineeResearch Network shown the perilous nature of button batteries, particularly when lodged in the esophagus, where they can cause rapid damage due to electrochemical reactions (16, 17).

Interestingly, plastic materials such as lollipop sticks and in-game currency were also noted, aligning with findings by Cho et al. (18), who noted that although plastic objects are less radiopaque and harder to detect, they still constitute a significant proportion of ingested foreign bodies, especially in the younger age groups. While sharp objects such as fish bones and hairpins have been documented in the literature (15), their appearance in our study (10 cases) was less frequent, highlighting the somewhat unpredictable nature of sharp object ingestion. Sharp objects, particularly when they enter the gastrointestinal tract, pose a substantial risk of perforation, obstruction, or bleeding, underscoring the importance of early recognition and management (19).

The anatomical distribution of foreign bodies in our cohort aligns with previous studies (11, 20), with the stomach being the most common site of lodging (42.2%), followed by the upper esophagus (34.86%). The upper esophagus is frequently affected by coins and other metal objects, likely due to their size and shape. These objects often get trapped in the upper esophageal sphincter, causing immediate obstruction and potentially compromising respiratory function. Conversely, batteries and plastic objects were more commonly found in the stomach. The varied material types found in the stomach underscore the importance of considering the entire spectrum of potential foreign bodies when developing management plans. In comparison, studies (21, 22) have similarly found that the stomach and upper esophagus are the most frequent sites for foreign body lodging in pediatric patients. The lower esophagus accounted for only 10.09% of cases in our study, suggesting that while it is a possible location for foreign body impaction, it is less common due to the physiological anatomy of the esophagus, which prevents objects from passing further down.

Complications arising from foreign body ingestion were observed in 43% of our cohort, with congestion, edema, and erosion being the most frequent findings. These complications align with studies (2, 23), which identified congestion and erosion as the most common complications. The presence of congestion and edema in patients who ingested larger or sharper objects, like hairpins or fish bones, which can lead to secondary infections, bleeding, or long-term esophageal stricture. The combination of congestion, edema, and erosion (12 cases) points to more significant injury and further complicates management, as these children are at greater risk for long-term complications such as stricture formation or esophageal perforation. A study (24) also highlighted that the presence of multiple complications after FBI ingestion necessitates a more aggressive and multi-faceted treatment approach, involving both medical and surgical intervention.

In our cohort, the majority of foreign bodies were removed using foreign body forceps, with fewer cases requiring alternative methods such as snares, baskets, or loop extractors. The use of forceps and snares for removal is well-documented in the literature (25), where they are considered the first-line tools for removing objects lodged in the esophagus and stomach. Magnetic beads, a particularly dangerous foreign body type due to their tendency to attract to each other, were removed using specialized tools like magnets. The effectiveness of these methods aligns with the previous studies (26), who noted that endoscopic removal is the most effective method.

One of the limitations of this study is the retrospective nature of the data, which may have introduced biases due to incomplete or inconsistent reporting. Indeed, the persistence of coin ingestion in the post-COVID era is an interesting observation in our study. However, we are not able to formally assess temporal changes in FBI patterns over time. The definition of complications in the present analysis was relatively narrow and did not include some clinically important outcomes, such as gastrointestinal perforation, fistula formation, stricture, or delayed injury. Detailed time-to-intervention data for endoscopic foreign body removal were not consistently available, as the interval between ingestion and presentation varied substantially. Future prospective studies, ideally multicenter, could provide more robust data on the effectiveness of different removal techniques and complications associated with specific materials. Additionally, while the study provides useful insights into the most common foreign body types and locations, it does not delve deeply into the long-term outcomes or potential delayed complications, such as stricture formation or esophageal motility disorders, which could be important areas for future research.

## 5 Conclusion

This study provides valuable insights into the prevalence, material types, anatomical locations, and complications associated with foreign body ingestion in pediatric patients. The findings emphasize the need for timely diagnosis and intervention to prevent severe complications. The high incidence of foreign body ingestion in younger children, particularly boys, along with the predominance of metal objects and the frequent involvement of the upper esophagus and stomach, offers crucial insights for clinical practice. Further prospective, multicenter studies are needed to refine management strategies and improve outcomes in pediatric patients with foreign body ingestion.

## Data Availability

The original contributions presented in the study are included in the article/Supplementary Material, further inquiries can be directed to the corresponding author/s.
